# Chemokine receptor CXCR4 expression in hepatocellular carcinoma patients increases the risk of bone metastases and poor survival

**DOI:** 10.1186/1471-2407-9-176

**Published:** 2009-06-09

**Authors:** Zuo-lin Xiang, Zhao-chong Zeng, Zhao-you Tang, Jia Fan, Peng-yuan Zhuang, Ying Liang, Yun-shan Tan, Jian He

**Affiliations:** 1 Department of Radiation Oncology, Zhongshan Hospital, Fudan University, Shanghai, PR China; 2 Liver Cancer Institute, Zhongshan Hospital, Fudan University, Shanghai, PR China; 3 Department of Pathology, Zhongshan Hospital, Fudan University, Shanghai, PR China

## Abstract

**Background:**

The chemokine and bone marrow-homing receptor CXCR4 is implicated in metastases of various cancers. This study was conducted to analyze the association of CXCR4 expression with hepatocellular carcinoma (HCC) bone metastasis and patient survival.

**Methods:**

Tumor tissue from HCC patients with (n = 43) and without (n = 138) bone metastasis was subjected to immunohistochemical staining for CXCR4 using tissue microarrays. Immunoreactivity was evaluated semi-quantitatively. A receiver-operating characteristic-based approach and logistical regression analysis were used to determine the predictive value of clinicopathologic factors, including CXCR4 expression, in bone metastasis. Patient survival was analyzed by Kaplan-Meier curves and log-rank tests.

**Results:**

CXCR4 overexpression was detected in 34 of 43 (79.1%) patients with bone metastases and in 57 of 138 (41.3%) without bone metastases. CXCR4 expression correlated with (correlation coefficient: 0.551, *P < 0.001) and was *predictive of HCC bone metastases (AUC: 0.689; 95%CI: 0.601 – 0.776; *P < 0.001*). CXCR4 staining intensity correlated with the bone metastasis-free survival (correlation coefficient: -0.359; P = 0.018). CXCR4 overexpression in primary tumors (n = 91) decreased overall median survival (18.0 months vs. 36.0 months, *P *<*0.001*). Multivariable analysis identified CXCR4 as a strong, independent risk factor for reduced disease-free survival (relative risk [RR]: 5.440; *P *= 0.023) and overall survival (RR: 7.082; *P *= 0.001).

**Conclusion:**

CXCR4 expression in primary HCCs may be an independent risk factor for bone metastasis and may be associated with poor clinical outcome.

## Background

Chemokines are chemotactic factors that regulate the development and migration of various cell types. These factors are classified into four groups (CXC, CX3C, CC, and C) based on the position of the first two highly conserved cysteines (C) in the amino acid sequence (where X is any amino acid residue). The chemical effects of chemokines on target cells are mediated by G protein-coupled receptors (GPCRs) that contain seven transmembrane domains and participate in attracting leukocytes to different organs [1]. The chemokine receptor CXCR4 is a 352-amino acid rhodopsin-like GPCR belonging to a large superfamily of GPCRs. CXCR4 selectively binds the CXC chemokine, stromal cell-derived factor 1 (SDF-1). SDF-1 secretion by stromal cells attracts cancer cells via stimulation of the CXCR4 receptor, which is upregulated in tumor cells. Binding of SDF-1 to CXCR4 induces migration of cancer cells into normal tissue, where the cells proliferate and form metastatic tumors [2]. Schimanski and colleagues [3] found that dissemination of hepatocellular carcinoma (HCC) may be mediated via the chemokine receptor CXCR4, but the relationship between CXCR4 expression and bone metastases of HCC remains unknown. Recent studies reveal that CXCR4 overexpression may be associated with bone or bone marrow metastasis and correlated with poor survival in various solid malignancies [4-11]. Blocking the CXCR4/SDF-1 pathway may prevent the formation of bone metastases [12].

In the past, bone metastases were considered uncommon in patients with HCC. However, due to improved duration of control of intrahepatic primary tumors and due to improved imaging, bone metastases from HCC are now more frequently noted [13,14]. Early diagnosis of bone metastasis is important for the therapeutic regimen and for assessing prognosis. HCC patients with bone metastases not only have a poor prognosis, but also suffer from pain and other significant symptoms that are detrimental to quality of life. Prevention of such bone metastases will depend on a full understanding of the molecular mechanisms that underlie HCC metastases to bone.

We hypothesize that CXCR4 participates in HCC cell homing to bone or bone marrow and that primary tumor CXCR4 expression correlates with clinical metastasis to bone. In this study, we examined the role of high CXCR4 expression levels in HCC bone metastasis and survival.

## Methods

### Patients

From May 1999 to October 2007, 43 patients with pathologically proven HCC underwent hepatectomy at the Liver Cancer Institute, Fudan University. All patients had bone metastases and complained of pain in the days following the hepatectomy. All patients received external-beam radiation therapy for their bone lesions at the Department of Radiation Oncology, Zhongshan Hospital, Fudan University. During the same time, 138 patients with pathologically proven HCC who underwent hepatectomy but did not have bone metastasis were retrieved from a prospectively designed database. Of the 138 patients, 25 patients had distal metastases but without bone involvement and 113 patients were without metastasis. This study was approved by the ethical review board of Zhongshan Hospital, Fudan University. The patient population consisted of 156 men and 25 women with a mean age of 50.7 ± 10.4 years (range: 12 – 86 years). Among these patients, 37 men and 6 women had bone metastases, and these patients had a mean age of 50.4 ± 9.5 years (range: 32 – 69 years). Tumor size was based on the largest dimension of the tumor. Satellite lesions were defined by the presence of two or more nodules, including intrahepatic metastases. Vascular invasion was determined by microscopic examination of the resected specimen. Portal vein thrombosis was identified by macroscopic examination of the resected specimen. Of the 181 HCC patients, 144 were positive for the hepatitis B surface antigen. All patients had Child-Pugh A liver function.

Diagnosis of bone metastases was based on the history of HCC, presence of symptoms, and radiologic imaging studies. Bone metastases were not confirmed by histological testing. Patients with primary lesions or seeding neoplasm invading bone were excluded from this study. All patients were screened by technetium-99m bone scintigraphy for possible bone metastasis. Bone scintigraphy is not specific for metastatic disease, and positive findings must be confirmed using other imaging studies. A confirmatory study (MRI, as a first choice, or CT) is obligatory in this study and is especially important to determine the presence or absence of soft-tissue extension with bone destruction or spinal cord compression as well as the extent of osteolytic or osteoblastic metastases.

### Tissue Microarray

**Figure 1:**
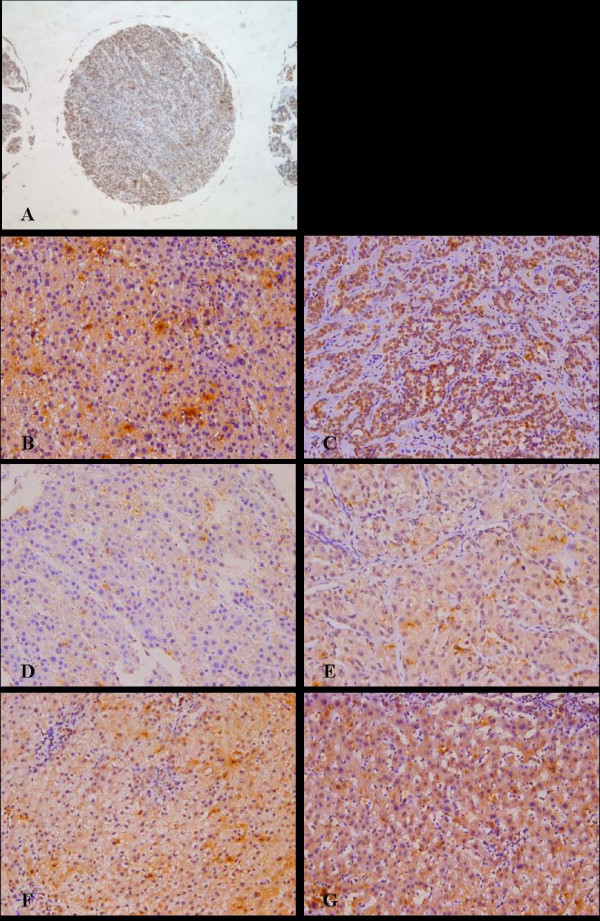
**CXCR4 expression in HCC TMAs**. Representative CXCR4 staining. (A): CXCR4 staining in a single tissue core with a 1-mm diameter (× 50). (B): Cytoplasmic CXCR4 staining. (C): Nuclear CXCR4 staining. (D-G): Examples of weak (+) (D), moderate (++) (E), strong (+++) (F), and very strong staining (++++) (G). All images are × 200 magnification unless otherwise noted.

The tissue microarray (TMA) technique was performed as described elsewhere [15]. Hematoxylin and eosin-stained slides were screened to identify optimal tumor tissue for analysis. TMA slides were then constructed (in collaboration with Shanghai Biochip Company, Ltd, Shanghai, China). Two cores of tissue were collected from non-necrotic areas of tumor foci (as shown in Figure 1A) in each formalin-fixed, paraffin-embedded HCC sample. Cores were collected with punch cores with a longest dimension of 1.0 mm. Sections (4 μm) of the resulting TMA blocks were made using standard techniques. Sufficient tumor tissue was available and analyzed in all 181 cases.

### Immunohistochemistry

Immunohistochemistry was performed with a two-step method using primary antibody and heat-induced antigen-retrieval procedures. Mouse monoclonal antibody to human CXCR4 served as the primary antibody (Cat. No. MAB172, Clone 44716, R & D Systems, Minneapolis, MN, USA). Tissue was incubated with anti-CXCR4 antibody (1:1,000) overnight at 4°C. The primary antibody was then removed, and the components of the Envision-plus detection system were applied, along with a polymer-linked anti-mouse antibody (EnVision+/HRP/Mo, Dako, Glostrup, Denmark). Reaction products were visualized by incubation with 3, 3'-diaminobenzidine. Sections were then counterstained with hematoxylin, dehydrated, and mounted.

### Scoring of CXCR4 Expression

Slides were examined by three independent, blinded observers using a Leica DMIRE2 microscope (Leica DMIRE2, CCD: DFC 420FX, Germany). The intensity of staining and percentage of positive tumor cells were determined by each observer, and the average of three scores was calculated. The intensity of staining (brown color) was scored semi-quantitatively as follows: +, weak; ++, medium; +++, strong; and ++++, very strong. Samples receiving a score of ++ or greater were considered CXCR4-positive [16]. An overall immunostaining score was calculated by multiplying the percentage of positive tumor cells (0 – 100) by the staining intensity (Grade 1 – 4), producing a score in the range of 0 – 400 [17].

### Follow-Up Assessments

Follow-up assessments were performed at 3-month intervals after hepatectomy. At every visit a history was collected, and a physical examination was performed. Chest radiography and ultrasonographic examination of the liver were performed at 6 months. Bone scintigraphy was performed when the patient complained of pain. Laboratory tests (liver function, α-fetoprotein, and routine blood tests) were carried out every 3 months. The median follow-up times of HCC patients with and without bone metastasis were 20.0 months and 23.5 months, respectively.

### Statistical Analysis

Patients' overall survival (OS) was defined by the interval from the date of hepatectomy to the date of death regardless of cause or the time elapsed since the last follow-up appointment. Disease-free survival (DFS) was calculated from the date of operation to the date of recurrence, metastasis, or death. Bone metastasis-free survival was calculated from the date of surgery to the date of bone metastasis or death. All calculations were performed with SPSS 15.0 for Windows. Pearson χ^2 ^test or Fisher exact test was used to compare qualitative variables. Quantitative variables were analyzed using the Student's *t *test. Logistical regression analysis and receiver operating characteristic (ROC) curves were used to determine the predictive value of parameters. Kaplan-Meier analysis was used to determine OS, DFS, and bone metastasis-free survival. Survival outcomes between patient subgroups were compared using the log-rank test. Multivariate analysis with the Cox regression model was performed to identify the clinicopathologic factors that independently predicted survival. Probability values of less than 0.05 were considered significant.

## Results

### Clinicopathologic Characteristics

Statistical analysis of HCC patients with bone metastases and without metastases revealed that these two groups significantly differed in the presence of satellite lesions, margin status, vascular invasion, UICC T stage, Edmondson grade, and CXCR4 status. CXCR4 expression was higher in patients with bone metastases than in those with distal metastases, but without bone involvement (Table 1).

**Table 1:** Clinicopathologic factors related to bone metastases in 181 HCC patients

		HCC Bone Metastases			
			
Clinicopathologic Parameters	No. of Cases	Negative (n = 138)			*P *Value (*P*_**1**_, *P*_**2**_)§
				
		A* (n = 113)	B* (n = 25)	Positive (n = 43)	
**Age (y)**					
≤ 60	153	94 (83.2%)	21 (84.0%)	38 (88.4%)	*P*_1 _= 0.422
> 60	28	19 (16.8%)	4 (16.0%)	5 (11.6%)	*P*_2 _= 0.434
**Gender**					
Female	25	15 (13.3%)	4 (16.0%)	6 (14.0%)	*P*_1_= 0.912
Male	156	98 (86.7%)	21 (84.0%)	37 (86.0%)	*P*_2 _= 0.540
**HBsAg **					
Negative	37	22 (19.5%)	5 (20.0%)	10 (23.3%)	*P*_1_= 0.601
Positive	144	91 (80.5%)	20 (80.0%)	33 (76.7%)	*P*_2 _= 0.755
**AFP **(ng/mL)	181				
≤ 20	48	28 (24.8%)	7 (28.0%)	13 (30.2%)	*P*_1_= 0.544
20 – 400	59	39 (34.5%)	9 (36.0%)	11 (25.6%)	*P*_2 _= 0.648
≥ 400	74	46 (40.7%)	9 (36.0%)	19 (44.2%)	
**Cirrhosis**					
Absence	45	30 (26.5%)	6 (24.0%)	9 (20.9%)	*P*_1_= 0.469
Presence	136	83 (73.5%)	19 (76.0%)	34 (79.1%)	*P*_2 _= 0.768
**Satellite Lesion**					
Absence	127	94 (83.2%)	13 (52.0%)	20 (46.5%)	*P*_1_< 0.001※
Presence	54	19 (16.8%)	12 (48.0%)	23 (53.5%)	*P*_2 _= 0.662
**Tumor Size (cm)**	181	6.04 ± 3.57	6.92 ± 3.34	7.26 ± 3.19	*P*_1_= 0.051 *P*_2 _= 0.676
**Margins**					
Clear	79	56 (49.6%)	10 (40.0%)	13 (30.2%)	*P*_1_= 0.030※
Involved	102	57 (50.4%)	15 (60.0%)	30 (69.8%)	*P*_2 _= 0.412
**Vascular invasion**					
Absence	95	70 (61.9%)	9 (36.0%)	16 (37.2%)	*P*_1_= 0.006※
Presence	86	43 (38.1%)	16 (64.0%)	27 (62.8%)	*P*_2 _= 0.921
**Portal Vein Thrombosis**					
Absence	153	97 (85.8%)	20 (80.0%)	36 (83.7%)	*P*_1_= 0.739
Presence	28	16 (14.2%)	5 (20.0%)	7 (16.3%)	*P*_2 _= 0.698
**Hilar lymph nodes**					
Absence	170	109 (96.5%)	23 (92.0%)	38 (88.4%)	*P*_1_= 0.066
Presence	11	4 (3.5%)	2 (8.0%)	5 (11.6%)	*P*_2 _= 0.488
**UICC T stage **					
T1	91	74 (65.5%)	7 (28.0%)	10 (23.3%)	*P*_1_< 0.001※
T2	38	17 (15.0%)	8 (32.0%)	13 (30.2%)	*P*_2 _= 0.856
T3	52	22 (19.5%)	10 (40.0%)	20 (46.5%)	
**Edmondson grade**					
Low (I/II)	118	81 (71.7%)	15 (60.0%)	22 (51.2%)	*P*_1_= 0.016※
High (III/IV)	63	32 (28.3%)	10 (40.0%)	21 (48.8%)	*P*_2 _= 0.481
**CXCR4**					
Negative	90	70 (61.9%)	11 (44.0%)	9 (20.9%)	*P*_1_< 0.001※
Positive	91	43 (38.1%)	14 (56.0%)	34 (79.1%)	*P*_2 _= 0.044※

* A: HCC without metastasis; B: Distal metastatic HCC without bone involvement.

§ *P*_1_: Statistical analysis of HCC patients with bone metastases (n = 43) and without metastases (n = 113).

*P*_2_: Statistical analysis of distal metastatic HCC without bone involvement (n = 25) and HCC patients with bone metastases (n = 43).

# Student *t *test.

※ Significant *P *value.

### CXCR4 Expression

Of the 181 tumor samples examined, 91 (50.3%) were positive for CXCR4 and 90 (49.7%) were negative. CXCR4 staining was detected in the cytoplasm (Figure 1B) and nucleus (Figure 1C), although it was primarily present in the former. Normal tissue adjacent to tumor cells occasionally showed weak CXCR4 staining in the cytoplasm. CXCR4 overexpression was detected in 34 of 43 (79.1%) patients with bone metastases and in 57 of 138 (41.3%) without bone metastases. Of the 138 patients without bone metastases, positive expression of CXCR4 was detected in 43 of 113 (38.1%) HCC patients without metastases and in 14 of 25 (56.0%) HCC patients with distal metastases but without bone involvement. In addition, CXCR4 expression much higher in patients with bone metastases than in those without metastases (*P < 0.001*) or those with distal metastases but without bone involvement (*P = *0.044). The overall CXCR4 immunostaining score (i.e., the product of the percent positive cells and staining intensity) was 210 ± 93.4 (median: 225; range: 49 – 400) in patients with bone metastases and 163 ± 91.0 (median: 176; range: 43 – 304) in those with distal metastases but without bone involvement (*P *= 0.048). Analysis of the association between CXCR4 expression and clinicopathological factors among the 181 HCC patients revealed that bone and hilar lymph node metastases were strongly associated with CXCR4 expression (Table 2). The extent of CXCR4 overexpression correlated with bone metastases (correlation coefficient: 0.551; *P *< 0.001), margin status (correlation coefficient: 0.206;*P *= 0.005), and hilar lymph node metastases (correlation coefficient: 0.198; *P *= 0.008).

**Table 2:** Clinicopathologic factors related to CXCR4 expression in 181 HCC patients

		CXCR4 Expression		
			
Clinicopathologic Parameters	No. of Cases	Negative (n = 90)	Positive (n = 91)	*P*
**Age (y)**				
≤ 60	153	76 (49.7%)	77 (50.3%)	0.975
> 60	28	14 (50.0%)	14 (50.0%)	
**Gender**				
Female	25	16 (64.0%)	9 (36.0%)	0.124
Male	156	74 (47.4%)	82 (52.6%)	
**HBsAg**				
Negative	37	22 (59.5%)	15 (40.5%)	0.184
Positive	144	68 (47.2%)	76 (52.8%)	
**AFP**(ng/mL, mean ± SD)	181	4941.25 ± 13475.41	3375.54 ± 11340.86	0.396
≤ 20	48	18 (37.5%)	30 (62.5%)	0.017*
20 – 400	59	26 (44.1%)	33 (55.9%)	
≥ 400	74	46 (62.2%)	28 (37.8%)	
**Cirrhosis**				
Absence	45	22 (48.9%)	23 (51.1%)	0.897
Presence	136	68 (50.0%)	68 (50.0%)	
**Satellite lesion**				
Absence	127	65 (51.2%)	62 (48.8%)	0.548
Presence	54	25 (46.3%)	29 (53.7%)	
**Tumor size (cm)**	181	6.15 ± 3.66	6.75 ± 3.28	0.247
**Margins**				
Clear	79	45 (57.0%)	34 (43.0%)	0.087
Involved	102	45 (44.1%)	57 (55.9%)	
**Vascular invasion**				
Absence	95	44 (46.3%)	51 (53.7%)	0.335
Presence	86	46 (53.5%)	40 (46.5%)	
**Portal Vein Thrombosis**				
Absence	153	72 (47.1%)	81 (52.9%)	0.094
Presence	28	18 (64.3%)	10 (35.7%)	
**Hilar Lymph Nodes**				
Absence	170	89 (52.4%)	81 (47.6%)	0.005*
Presence	11	1 (9.1%)	10 (90.9%)	
**UICC T stage**				
T1	91	45 (49.5%)	46 (50.5%)	0.693
T2	38	17 (44.7%)	21 (55.3%)	
T3	52	28 (53.8%)	24 (46.2%)	
**Edmondson grade**				
Low (I/II)	118	61 (51.7%)	57 (48.3%)	0.468
High (III/IV)	63	29 (46.0%)	34 (54.0%)	
**Bone Metastases**				
Absence	138	81 (58.7%)	57 (41.3%)	< 0.001*
Presence	43	9 (20.9%)	34 (79.1%)	

* Significant *P *value.

### Predictors of Bone Metastasis

**Figure 2:**
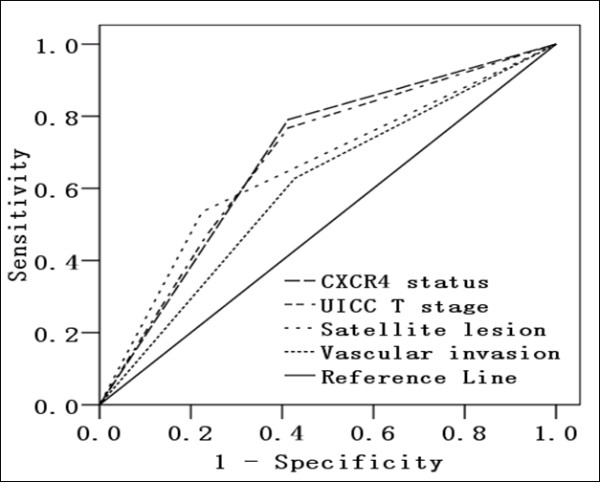
**ROC analysis**. ROC analysis of CXCR4, UICC T stage, satellite lesion, and vascular invasion. The AUC of all parameters was > 0.5, indicating that they were predictive of HCC bone metastases.

Bone metastases from HCC correlated with UICC T stage (correlation coefficient: 0.290, P < 0.001), satellite lesions (correlation coefficient: 0.289, P < 0.001), vascular invasion (correlation coefficient: 0.171, P = 0.022), and margins (correlation coefficient: 0.151, P = 0.042). In addition, bone metastases strongly correlated with CXCR4 expression and staining intensity (correlation coefficient: 0.551, P < 0.001). ROC curve analysis was performed for these clinicopathological factors. The area under the curve (AUC) and the 95% confidence interval (CI) were used to assess the power of these clinicopathological factors for predicting bone metastases (Figure 2 and Table 3). ROC analysis confirmed that CXCR4 status had good accuracy in predicting bone metastases (AUC: 0.689; 95%CI: 0.601 – 0.776; *P < 0.001*). However, CXCR4 expression did not correlate with the number of bone metastases (P = 0.832) or the site of bone metastases (P = 0.104).

**Table 3:** Area under the curve (95% Confidence Interval) demonstrating the discriminatory power of each clinicopathologic factor for predicting HCC bone metastases

Clinicopathologic Factor	AUC	SE	95%CI	*P*
**CXCR4 Status**	0.689	0.045	0.601 – 0.776	<0.001*
**UICCT Stage**	0.684	0.046	0.595 – 0.773	<0.001*
**Satellite Lesion**	0.655	0.050	0.557 – 0.753	0.002*
**Vascular Invasion**	0.600	0.049	0.504 – 0.697	0.048*
**Edmondson Grade**	0.593	0.049	0.498 – 0.688	0.066
**Margins**	0.588	0.049	0.492 – 0.683	0.082

* Significant *P *value

### CXCR4 Expression and Survival

**Figure 3:**
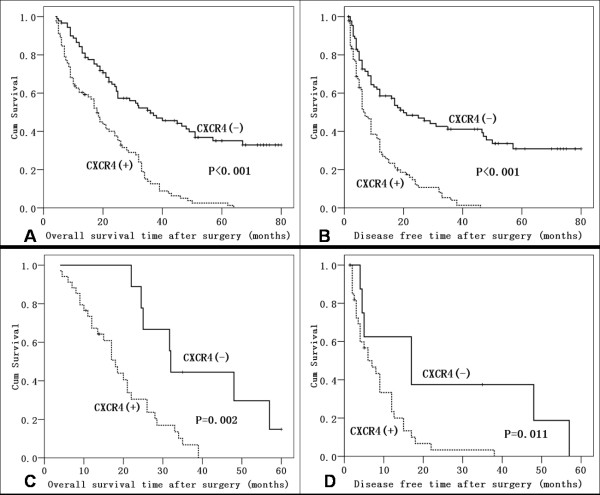
**Kaplan-Meier analysis of overall and disease-free survival**. (A and B): Association of CXCR4 expression with overall (A) and disease-free (B) survival of 181 HCC patients. (C and D): Association of CXCR4 expression with overall (C) and disease-free (D) survival of the 43 HCC patients with bone metastases.

Log-rank tests of Kaplan-Meier curves revealed that, after hepatectomy, among all 181 HCC patients, those with positive CXCR4 expression had a poorer OS and DFS (Log-rank *P *< 0.001, Figure 3A and 3B). Similar results were obtained for the 43 HCC patients with bone metastases (Figure 3C and 3D). CXCR4 expression level correlated with bone metastasis-free survival (correlation coefficient: -0.359, P = 0.018). Multivariate analysis identified CXCR4 expression (RR: 7.082; *P *= 0.001) and UICC T stage (*P *= 0.001) as the two factors that independently predicted survival of HCC patients with bone metastasis. CXCR4 (RR: 5.440; *P *= 0.023) and UICC T3 stage (*P *= 0.010) were the independent risk factors that most strongly associated with reduced disease-free survival in patients with bone metastasis.

## Discussion

HCC is the most common primary tumor of the liver. Once HCC cells spread to and become established in bone, HCC bone metastases begin to interfere with the normal health and strength of the bones. This often leads to bone pain, fracture, or other complications that can significantly impair a patient's health and that increase morbidity and mortality in HCC patients. The frequency of bone metastasis in HCC patients with extrahepatic metastases was estimated to be 38.5% [14]. Early prediction or detection of bone metastases can help to determine the best treatment strategy and avoid complications caused by the metastases.

The mechanisms of cancer metastasis are complex, leading to continual expansion of the "seed and soil" hypothesis upon new discoveries about this process. After metastatic cells have passed through vascular channels and implanted in the bone, a favorable "soil" may be responsible for further growth and proliferation. The establishment of bone metastasis results from close interaction between metastatic tumor cells and the unique environment of the bone and bone marrow [18]. The formation of aggressive bone metastases is tightly associated with a distinctive gene expression profile [19]. The chemokine receptor CXCR4 has been implicated in the mechanism underlying tumor cell metastasis to bone. The interaction between the organ microenvironment and cancer cells appears to be fundamental for establishing metastatic growth. Tumors with higher CXCR4 expression are more prone to clinical metastasis than those with lower expression [20,21].

Cancer stem cells express CXCR4 at their surface. As a result, the SDF-1/CXCR4 axis participates in directing the metastasis of these cells to bones that express high levels of SDF-1 [22]. High levels of SDF-1 are present in organs that are commonly invaded by metastasizing cells, such as the bones, and these high SDF-1 levels attract CXCR4-positive tumor cells. The interaction between SDF-1 and CXCR4 leads to the activation of specific signaling pathways, allowing for homing and metastatic progression. CXCR4 overexpression enhances bone tumor growth and osteolysis [23]. It also significantly decreases bone metastasis-free survival *in vivo *[24]. CXCR4 may enhance invasive signals and metastatic growth in the bone microenvironment.

Here, we found that high CXCR4 expression in HCC specimens is clinically correlated with bone metastases. CXCR4 may play an important role in the metastasis of HCC by promoting the migration of tumor cells [25]. CXCR4-overexpressing cells were more likely to metastasize, notably into bone marrow [26]. Prostate cancers, and perhaps other neoplasms, may rely upon the SDF-1/CXCR4 pathway to spread to bone [5]. Our findings suggest that HCC also uses the same pathway to spread to bone.

Using a ROC-based approach and logistical regression analysis to select the most relevant factors from a panel of clinicopathologic factors, we found that CXCR4, UICC T stage, satellite lesions, and vascular invasion were predictors of bone metastases from HCC. CXCR4 expression, in particular, was a superior predictor of bone metastasis and increased in primary HCC tumors with clinical evidence of bone metastases. These results are consistent with our hypothesis that CXCR4 is important for HCC cell homing to bone or bone marrow, and they suggest that CXCR4 overexpression in HCC is associated with a higher rate of bone metastases. Here, we report for the first time that overexpression of CXCR4 is significantly correlated with HCC bone metastases and that positive expression of CXCR4 may reduce HCC patient survival. We also found that the level of CXCR4 expression correlated with bone metastasis-free survival; i.e., HCC patients with tumor tissue exhibiting strong CXCR4 expression may experience bone metastases sooner than those with lower levels of expression. Smith and colleagues found that CXCR4 inhibitors improve treatment of patients with primary and metastatic cancer [27]. Thus, blocking the CXCR4/SDF-1 pathway would be expected to prevent bone metastases. The patients whom we collected for this study are not consecutive patients, which may affect our conclusions.

During the early establishment of metastases, bone is destroyed by osteoclasts. Bisphosphonates can inhibit the activity of osteoclasts, and bisphosphonate treatment is a well-established supportive therapy for reducing the frequency and severity of skeletal complications in patients with bone metastases from different cancers [28]. Bisphosphonates, such as clodronate, are potent inhibitors of bone resorption. Clodronate has been used for many years to reduce skeletal complications of bony metastases and also has potential for inhibiting bone metastasis. Breast cancer patients who receive a 2-year treatment of clodronate have a significantly decreased occurrence of bone metastases compared to patients receiving placebo [29]. Diel and coworkers [30] also found the incidence of osseous metastases was significantly lower in the clodronate-treated breast cancer patients than in control patients. Our findings suggest that HCC patients at high risk of bone metastases can be identified by analyzing CXCR4 expression and other clinicopathological factors. Once identified, these high-risk HCC patients could receive oral clodronate, which may reduce the risk of bone metastases.

In this study, we found that some HCC patients exhibited high levels of of CXCR4 expression but did not have bone metastases. This may be attributable to low levels of SDF-1 in the bones or bone marrow, a condition that would block the CXCR4/SDF-1 pathway. However, we did not measure SDF-1 levels in the bone of HCC patients. Determining whether SDF-1 levels predict bone metastasis in HCC patients will require further study. In addition, whether plasma levels of SDF-1 correlate with SDF-1 levels in the bone or bone marrow should be determined. In this way, analysis of CXCR4 expression and plasma SDF-1 may be combined to more accurately predict bone metastasis of HCC.

In summary, the clinical findings from this study strongly support a homing or signaling mechanism for HCC bone metastasis. In HCC, the CXCR4 chemokine receptor may facilitate metastasis to the bone, where the CXCR4 ligand is abundantly produced. CXCR4 is novel prognostic marker for bone metastases after curative resection of HCC. CXCR4 may not only prove useful for predicting bone metastasis, but may also serve as a therapeutic target for HCC.

## Conclusion

Our study results showed that CXCR4 expression significantly correlated with bone metastasis in HCC. CXCR4 overexpression in primary HCCs may be an independent risk factor for bone metastasis and may be associated with poor clinical outcome. CXCR4 may serve as a therapeutic target for HCC.

## Competing interests

The authors declare that they have no competing interests.

## Authors' contributions

ZCZ organized the study, planned the experiments, performed the statistical analysis and helped to write the manuscript. ZLX contributed to the design of the study, performed the statistical analysis, and drafted the manuscript. ZYT and JF participated in the design and coordination of the study. PYZ, YL, and YST contributed to the interpretation of the immunohistochemical data. JH selected the samples. All authors read and approved the final manuscript.

## Pre-publication history

The pre-publication history for this paper can be accessed here:

http://www.biomedcentral.com/1471-2407/9/176/prepub
